# An NMR relaxometry approach for quantitative investigation of the transchelation of gadolinium ions from GBCAs to a competing macromolecular chelator

**DOI:** 10.1038/s41598-021-00974-4

**Published:** 2021-11-05

**Authors:** Patrick Werner, Matthias Taupitz, Leif Schröder, Patrick Schuenke

**Affiliations:** 1 Molecular Imaging, Leibniz-Forschungsinstitut für Molekulare Pharmakologie (FMP), Berlin, Germany; 2 Department of Radiology, Charité – Universitätsmedizin Berlin, corporate member of Freie Universität Berlin, Humboldt-Universität Zu Berlin, and Berlin Institute of Health, Berlin, Germany; 3 Division of Translational Molecular Imaging, Deutsches Krebsforschungszentrum (DKFZ), Heidelberg, Germany; 4 Physikalisch-Technische Bundesanstalt (PTB), Braunschweig, Berlin, Germany

**Keywords:** Translational research, Biophysical chemistry, Biopolymers in vivo

## Abstract

Gadolinium-based contrast agents (GBCAs) have been used in clinical Magnetic Resonance Imaging (MRI) for more than 30 years. However, there is increasing evidence that their dissociation in vivo leads to long-term depositions of gadolinium ions in the human body. In vitro experiments provide critical insights into kinetics and thermodynamic equilibria of underlying processes, which give hints towards the in vivo situation. We developed a time-resolved MRI relaxometry-based approach that exploits distinct relaxivities of Gd^3+^ in different molecular environments. Its applicability to quantify the transmetallation of GBCAs, the binding of Gd^3+^ to competing chelators, and the combined transchelation process is demonstrated. Exemplarily, the approach is applied to investigate two representative GBCAs in the presence of Zn^2+^ and heparin, which is used as a model for a macromolecular and physiologically occurring chelator. Opposing indirect impacts of heparin on increasing the kinetic stability but reducing the thermodynamic stability of GBCAs are observed. The relaxivity of resulting Gd-heparin complexes is shown to be essentially increased compared to that of the parent GBCAs so that they might be one explanation for observed long-term MRI signal enhancement in vivo. In forthcoming studies, the presented method could help to identify the most potent Gd-complexing macromolecular species.

## Introduction

Magnetic resonance imaging (MRI) is a widely used non-invasive diagnostic imaging modality that is an indispensable tool in both clinical routine and experimental research. A significant fraction of the examinations (> 50% of all clinical exams in 2009)^1^ is performed with intravenous injection of gadolinium-based contrast agents (GBCA) to improve diagnostic value. The global yearly consumption involves 50 tons of gadolinium (Gd)^2^ that has to be administered in a safe formulation. Most GBCAs originally approved for clinical use are low-molecular weight agents that mainly differ in the chemical structure of their chelators (linear vs. macrocyclic)^3,4^. They all have in common that the bound paramagnetic Gd ion shortens the observed relaxation times of surrounding protons^5,6^, which causes the desired signal enhancement and improved contrast in GBCA-enhanced MRI.

**Figure 1:**
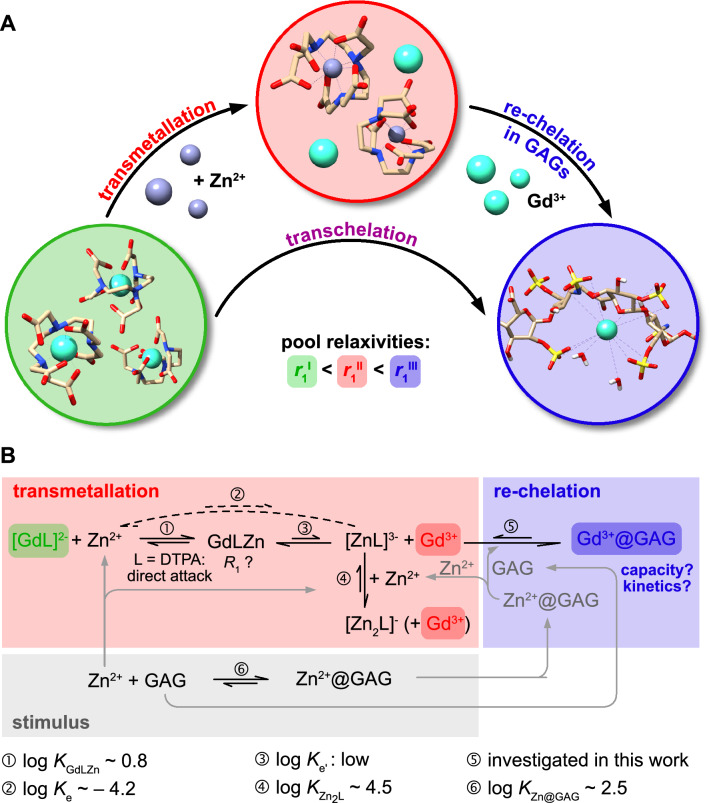
Stepwise zinc-induced rearrangements of chelated Gd in the presence of competing macromolecular chelators like the GAG heparin. The three distinct relaxivities for following Gd^3+^ through the process steps, *r*_1_^I^, *r*_1_^II^, *r*_1_^III^, are key to quantitative analysis. (**A**) In the transmetallation step, the central Gd^3+^ ion in the parent GBCA complex is replaced by Zn^2+^ (the intermediate Zn^2+^ complex is shown). Subsequently, the dissociated Gd^3+^ ions bind into the GAG structure. Coordination of the Gd^3+^ ion in the GAG structure is only schematic and was not calculated. (**B**) Details of the involved chemical equilibria. Zn^2+^ and GAG serve as stimulus where Zn^2+^ can also interact with the GAG matrix. For linear chelators, Zn^2+^ directly attacks the ligand L and initially forms a hetero dinuclear complex GdLZn as an intermediate with moderate stability. The transmetallation to ZnL with release of Gd^3+^ (no further additives) is thermodynamically unfavored (the dashed direct reaction does not occur but the net equilibrium constant *K*_e_ is known). Thus, the release of Gd from GdLZn must occur with low equilibrium constant *K*_e_‘. For efficient release of Gd^3+^ and to keep L blocked, the second intermediate ZnL + Gd^3+^ should be converted. To render the reaction irreversible and prevent Gd^3+^ binding to L, additional Zn^2+^ (provided directly or indirectly from the stimulus) can form the more stable Zn_2_L complex. Moreover, the GAG can serve to sequester Gd^3+^ and thus prevent re-formation of GdLZn. The parameters for equilibrium ➄ are unknown, i.e., the “capacity” of the GAG pool to scavenge Gd^3+^ as well as the time it takes to reach a new steady state. Equilibrium reaction arrows and stability constants are given only for qualitative orientation summarizing values at ~ 25 °C from (17, 36, 37).

Clearance from the body is a critical aspect of all injected agents. For GBCAs, it was assumed that they are renally excreted from the human body as unchanged compounds^7^. They have been considered safe and harmless for patients even after multiple administrations^8–12^. However, Gd chelates can be attacked, e.g., by competing ions like Zn^2+^, which can trigger new intermediates, and eventually cause an exchange with the central Gd ion—a process called transmetallation (Fig. 1a)^13,14^, which leads to the release of toxic Gd^3+^ ions^15,16^. Subsequently, endogenous substances might act as competing chelators or ligands for the released Gd ions. The transmetallation can include intermediate states of limited thermodynamic stability and the ligand L can be involved in formation of hetero- and homo-dinuclear complexes like GdLZn or Zn_2_L (see Fig. 1b)^17^.

The situation in vivo is complex because both Gd^3+^ and competing ions like Zn^2+^ may interact with various other endogenous binding partners that could play a significant role in sequestration of Gd^3+^ from its original chelator and thus push the chemical equilibrium away from the parent GBCA complex. This aspect is underexplored and requires quantitative in vitro studies using tools that enable the quantification of the transmetallation process and the subsequent re-binding of released Gd ions to potential competing chelators with regard to thermodynamic and kinetic aspects. While initial intermediates can have an unsuspicious low thermodynamic stability (e.g., (Zn^2+^)_1_-DTPA) and their relapse to the original GBCA complex is not unlikely, suitable stimuli can foster progression of the transmetallation and the kinetic stability upon such stimuli is an important factor.

Understanding the transmetallation and re-binding mechanisms is of high interest since first studies reported the detection of Gd deposition in the brain in 2013. Afterwards, many case studies have reported Gd depositions in a variety of organs including the brain^15,18–21^, skin^22,23^, bones^24^, eyes^25^, lung^23^, heart^26^, kidney^23^ and liver^27,28^. Based on the diversity of reported Gd deposition sites, it can be concluded that the competing endogenous chelators must be distributed almost throughout the whole human body and be easily accessible for dissociated Gd^3+^. Further, recent insights show that it must be macromolecular substances with high molecular weights (MW > 66.5 kDa) in order to explain the observed hyperintensities in vivo that are associated with the Gd deposition in tissue^29,30^.

Taupitz et al. could show that heparin, an endogenous sugar structure from the glycosaminoglycan (GAG) family, is a suitable candidate for such a complexing macromolecule^31^. The ability of GAGs to serve as chelators for a variety of ions had been proven before^31–35^ and unfractioned heparin, e.g., has a two-stage binding mechanism for Zn^2+^, which itself plays a crucial role in attacking linear chelators^36,37^. Competing chelators like GAGs could have an impact on shifting the equilibria towards a new steady state and in vitro models must be considered as references for delineating the boundary conditions when interpreting future in vivo data.

The goal of this study was to develop an NMR relaxometry approach for investigating and quantifying the combined influence of competing ions and competing macromolecular chelators on the stability of GBCAs. A further goal was to determine the capacity of endogenous chelators to sequester Gd^3+^ and to investigate whether time-resolved experiments provide new insights into the kinetic aspects of the underlying processes. The central idea is that the distinct relaxivities of Gd^3+^ in different environments (parent GBCA, free Gd^3+^, GAG-bound Gd^3+^) can be used to decompose and quantify the transmetallation of GBCAs, the binding process of Gd^3+^ ions to alternative chelators, as well as the combined transchelation process with sufficient temporal resolution. While the situation in vivo for tissue-bound Gd^3+^ can be more complex (rotational tumbling may differ from in vitro conditions), this should allow to identify the most effective competing ions leading to transmetallation, to identify the most potent Gd-complexing macromolecular species, as well as to investigate and distinguish thermodynamic and kinetic stability changes of the parent GBCA complexes.

At first, we used the approach to quantify the zinc-induced transmetallation process of two representative DOTA- and DTPA-based GBCAs. Competing Zn^2+^ ions were exemplarily compared to less potent Ca^2+^ ions. Afterwards, we applied the approach to quantify the binding capacity (for Gd^3+^ ions) of heparin, which was used as representative endogenous GAG. Furthermore, we exemplarily quantified the complete time-resolved zinc-induced transchelation process from both GBCAs to heparin. Altogether, the proposed NMR relaxometry-based approach allowed to decompose and quantify all three processes and provided important new insights into the transchelation process of Gd ions from GBCAs to heparin. Thus, it can probably serve as a valuable tool in forthcoming studies and provide meaningful contributions to current GBCA safety discussions.

## Results

### Relaxivity references

**Figure 2:**
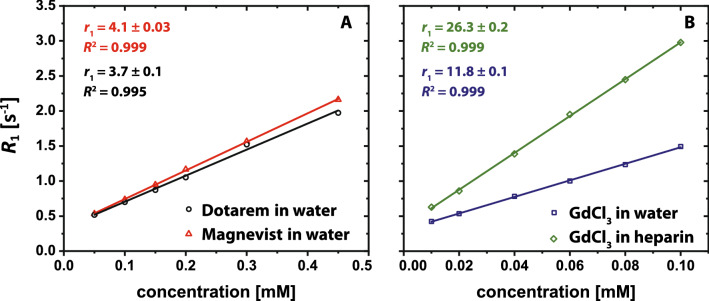
Relaxivity determinations at 9.4 T and 25 °C. (**A**) Relaxivity plots of Magnevist (red triangles) and Dotarem (black circles) in nanopure water. (**B**) Relaxivity plots of GdCl_3_ in nanopure water (blue squares) and in aqueous heparin solution (green diamonds; [heparin] = 100 µM). For each data point, the ROI-averaged mean value of ten independently acquired *R*_1_ maps was calculated. The shown relaxivity values are given by the slopes of the obtained linear fits. Please note the different concentration ranges in the subplots.

For later quantifications based on ^1^H *T*_1_ relaxometry, we determined the relaxivities of all individual compounds used in our well-defined model solutions. The relaxivity plots of Magnevist and Dotarem in water are shown in Fig. 2A. Figure 2B shows the relaxivity plots of GdCl_3_ in water and in heparin solution. The determined relaxivities at 9.4 T and 25 °C of Magnevist (*r*_1_ ≈ 4.1 s^-1^ mM^−1^) and Dotarem (*r*_1_ ≈ 3.7 s^-1^ mM^−1^) are about 3 times smaller compared to the relaxivity of GdCl_3_ in water (*r*_1_ ≈ 11.8 s^-1^ mM^−1^), which is again about 2.5 times smaller compared to the relaxivity of GdCl_3_ in heparin solution (*r*_1_ ≈ 26.3 s^−1^ mM^−1^). The relaxivity of ZnCl_2_ is about 4 orders of magnitude smaller compared to all other compounds. All determined relaxivities at 9.4 T in water at 25 °C and 37 °C are summarized in supplemental Table S1. Literature values at identical conditions are not available for all compounds, but the general trend of the individual relaxivity values is in agreement with previous studies^31,38,39^. Table S1 further includes the relaxivity values of all compounds in 100 µM heparin solution instead of nanopure water at 9.4 T and 25 °C and 37 °C. These were measured to exclude any potential impact of heparin on the determined relaxivities.

### Transmetallation

**Figure 3:**
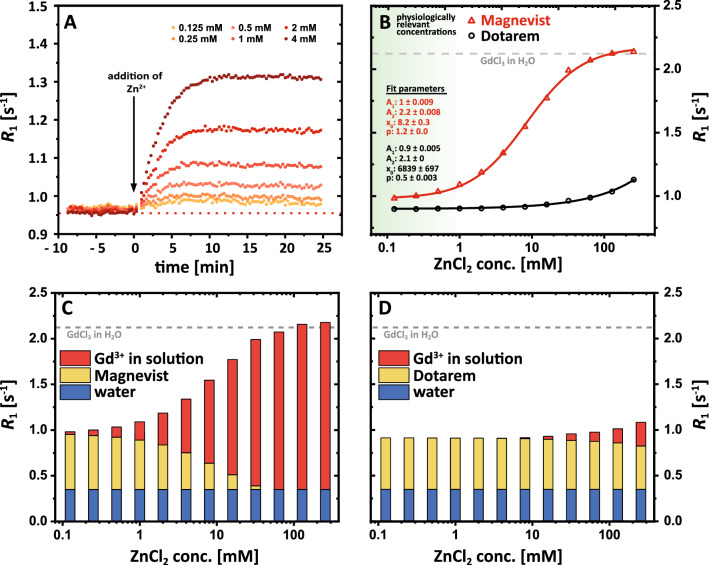
Results of transmetallation experiments at 9.4 T and 25 °C. (**A**) Time-resolved *R*_1_ relaxation rate measurements of 150 µM Magnevist in nanopure water after the addition of different ZnCl_2_ concentrations between 125 µM (yellow) and 4 mM (dark red). *R*_1_ values increase after the addition of ZnCl_2_ at *t* = 0 and reach new plateau values (time constant ≈ 2 min) depending on the ZnCl_2_ stimuli. (**B**) Final *R*_1_ values (new chemical equilibria) as a function of [ZnCl_2_] for Magnevist (red triangles) and Dotarem (black circles). The curve for Magnevist reaches a plateau value for [ZnCl_2_] > 100 mM, which matches the theoretical *R*_1_ value of 150 µM of GdCl_3_ (dashed gray line). The solid lines represent the fit using a logistic function. The fit results for both fits are displayed (*A*_2_ for Dotarem was fixed to the value of the dashed gray line). The physiological range of Zn^2+^ concentrations is labelled in green. (**C**) Bar charts of the individual contributions of water (blue), of the intact GBCA (yellow), of ZnCl_2_ (crosshatched), and of dissociated Gd^3+^ ions (red) to the overall observed relaxation rate of 150 µM Magnevist solution for different ZnCl_2_ concentrations. (**D**) Same as (**C**) but for Dotarem instead of Magnevist.

Using the determined relaxivities and Eqs. (1) and (2) (see methods section), the amount of Gd^3+^ ions that are released from a GBCA due to the transmetallation can be quantified. The measured relaxation rate (*R*_1_) of 150 µM Magnevist solution as a function of time is shown in Fig. 3A for six different ZnCl_2_ stimuli between 0.125 and 4 mM. The determined time constants for the transmetallation process at the ligand L (GdL to Zn_*i*_L, *i* = 1, 2) increase with increasing concentrations of ZnCl_2_ and vary between 1.34 min and 2.42 min for the investigated concentration range (Table 1). Hence, the observed pseudo-first order rate constant decreases for higher [Zn^2+^]. The final *R*_1_ values after transmetallation (plateaus of the six curves) increase with increasing ZnCl_2_ concentrations. This is illustrated in Fig. 3B, which shows the equilibrium *R*_1_ as a function of a very broad range of ZnCl_2_ concentrations between 0 and 256 mM. For Magnevist, a plateau is reached for ZnCl_2_ concentrations higher than ~ 100 mM (i.e., for a ratio ZnCl_2_:GBCA > 670). The plateau value (${R}_{1}\approx 2.1\, {\text s}^{-1}$) is similar to the expected *R*_1_ value of the full equivalent of free (but fully hydrated) Gd^3+^ from 150 µM of GdCl_3_. For Dotarem, no plateau value is reached as the mechanism of action is expected to differ from direct attack of Zn^2+^ onto the GdL complex; *R*_1_ values still increase for the highest concentration of 256 mM. Fitting these data using a logistic function (Fig. 3B, solid lines) allows determining characteristic values, which enable a quantitative comparison of stabilities. In the presented exemplary case, the ZnCl_2_ concentrations needed to dissociate 50% of the parent GBCA structures were determined to be about (8.2 ± 0.3) mM and (6839 ± 697) mM for Magnevist and Dotarem, respectively. This corresponds to a ca. 54-fold and 45,500-fold excess of zinc compared to the initial GBCA concentration of 150 µM. Similarly, the exemplary quantification for Ca^2+^ instead of Zn^2+^ is shown in supplementary Fig. S1. In agreement with previously reported stability constants of both divalent ions with various ligands, we found the calcium-induced transmetallation to be much less efficient^13,16^.

**Table 1:** Determined time constants for the dynamic transmetallation (τ in H_2_O) and dynamic transchelation (τ in heparin) experiments.

ZnCl_2_ (mM)	τ in H_2_O (min)	τ in heparin (d)
0.125	1.34 ± 0.01	> 21
0.25	1.42 ± 0.01	> 21
0.5	1.56 ± 0.01	21 ± 1
1	1.92 ± 0.01	3.04 ± 0.02
2	2.31 ± 0.01	0.41 ± 0.01
4	2.42 ± 0.01	0.074 ± 0.001

The values for τ in heparin for 0.125 mM and 0.25 mM ZnCl_2_ could not be fitted reliably and were thus just estimated to be larger than the 21 days as determined for 0.5 mM ZnCl_2_.

Solving Eq. (2) for every measured *R*_1_ value and employing Eq. (1) allows separating the individual contributions of water (${R}_{1,{\mathrm{H}}_{2}\mathrm{O}}$), of the intact GBCA (${R}_{1,\mathrm{GBCA}})$, of zinc chloride (${R}_{1,\mathrm{ZnCl}_{2}}$), and the released Gd^3+^ ions (${R}_{1,{\mathrm{Gd}}^{3+}})$ to the overall observed relaxation rate. These contributions are illustrated as bar charts for Magnevist (Fig. 3C) and Dotarem (Fig. 3D). With increasing ZnCl_2_ concentrations, the contribution of intact GBCA is decreasing, while the contribution of released Gd^3+^ ions is increasing. This effect is much more pronounced and starts for lower (~ 100-fold) ZnCl_2_ concentrations for Magnevist compared to Dotarem.

### Binding of gadolinium to heparin

**Figure 4:**
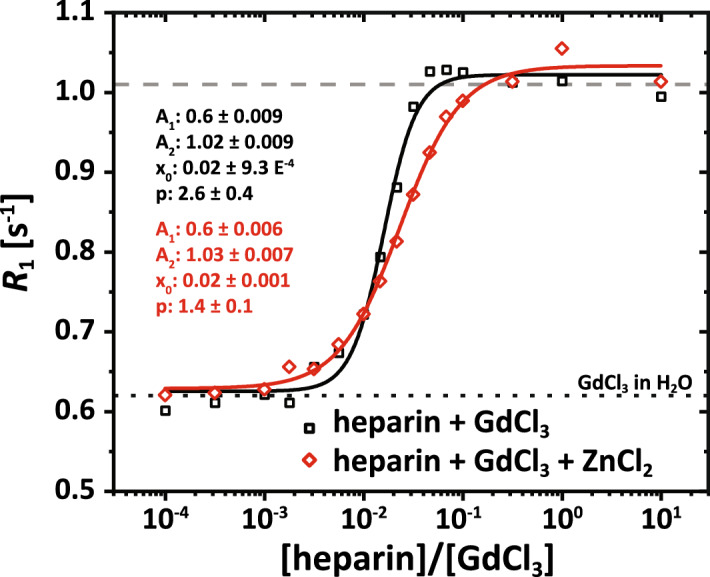
*R*_1_ titration curve of 25 µM GdCl_3_ with different heparin concentrations (c.f. supplemental Table S2) in the absence (black squares) and presence (red diamond) of ZnCl_2_ (833 µM) at 9.4 T and 25 °C. Both curves start at ${R}_{1}\approx 0.6 \, \mathrm{s}^{-1}$, which matches the expected relaxivity of 25 µM of free GdCl_3_ in H_2_O (dotted black line). For increasing concentration ratios, *R*_1_ of both curves increases until approaching a new plateau value of ${R}_{1}\approx 1.02 \, \mathrm{s}^{-1}$ at concentration ratios of about 10^–1^ and 1 in the absence and presence of ZnCl_2_, respectively. Beyond this point, all Gd^3+^ ions are bound to heparin and no free gadolinium remains in solution. One data point (red curve; ratio = 1) was excluded from the fit (logistic function) due to a presumable fault in the sample preparation.

Prior to the quantification of the complete transchelation process, we investigated the ability of the proposed approach to quantify the binding of Gd^3+^ ions to competing chelators. We used the method to exemplarily determine the capacity of a given heparin concentration for retaining Gd^3+^ ions by measuring *R*_1_ for different concentration ratios of heparin and GdCl_3_ (Fig. 4). The experiments were performed in the absence and presence of ZnCl_2_ (833 µM). The different concentration ratios between 10^–4^ and 10^1^ (c.f. supplemental Table S3) were realized by keeping a constant GdCl_3_ concentration of 25 µM while increasing the heparin concentration from 2.5 to 250 µM (based on average MW). Both curves start at a level where they match the expected relaxivity of 25 µM of GdCl_3_ in H_2_O (${R}_{1}\approx 0.63 \,\mathrm{s}^{-1}$). *R*_1_ asymptotically approaches a plateau of ${R}_{1}\approx 1.02\, \mathrm{s}^{-1}$ in both cases. This value matches the expected one (c.f. Fig. 2B) of 25 µM GdCl_3_ in heparin solution (dashed gray line). Like in the transmetallation scenario, fitting the data over the applied wide concentration range enables determining characteristic values like the point of inflection (PoI) and the slope at this point. The determined PoIs are very similar and occur for *x* ≈ 0.02 in the absence and presence of ZnCl_2_. Regarding the sequestration capacity for Gd at this point, the average molecular heparin unit binds ca. 22–31 Gd^3+^ ions (1/(2*x*)), depending on the presence of Zn^2+^. While the impact of Zn^2+^ seems to be minor for low heparin concentrations (a 33-fold excess shifts the PoI only marginally), the overall *R*_1_ transition with ZnCl_2_ appears flattened due to competition between diamagnetic Zn^2+^ and paramagnetic Gd^3+^ ions for the heparin binding sites. Quantitatively, this is described by the reduced value of fit parameter *p* in the presence of ZnCl_2_ (*p* = 1.4 ± 0.1) compared to *p* = 2.6 ± 0.4 in the absence of ZnCl_2_ (Fig. 4). In the plateau (x > 0.3), all present Gd^3+^ ions seem to be bound to heparin and no free Gd^3+^ ions remain in solution. Importantly, this justifies neglecting the term ${R}_{\mathrm{1,Gd\, in\, solution}}$ in Eq. (3) for such sufficiently large amounts of heparin. The step size between the equilibrium values in Fig. 4 allows estimating ${r}_{1,\mathrm{Gd@heparin}}=\left(1.02-0.35\right) \mathrm{s}^{-1}/25 \,\mu \mathrm{M}\approx 26.8 \,{\mathrm{s}}^{-1}{\mathrm{mM}}^{-1}$, which agrees well with the determined relaxivity of GdCl_3_ in heparin solution (${r}_{1}\approx 26.3 \, \mathrm{s}^{-1}{\mathrm{mM}}^{-1}$; c.f. Fig. 2B).

### Progressive transchelation

**Figure 5:**
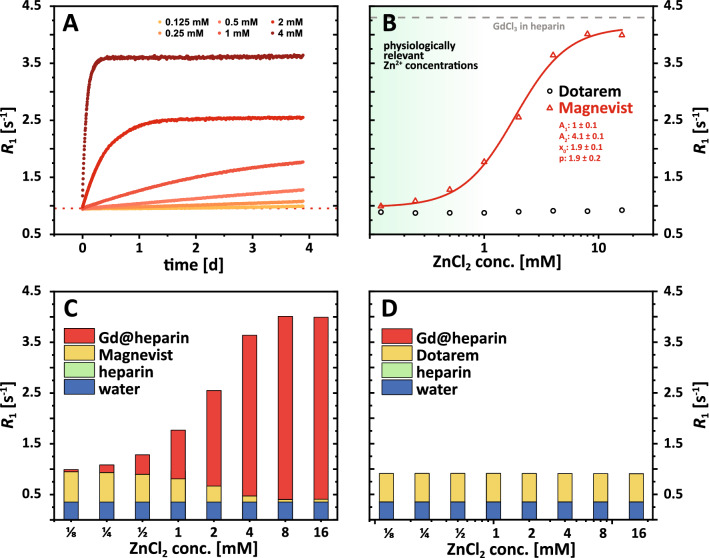
Results of transchelation experiments at 9.4 T and 25 °C. (**A**) Time-resolved *R*_1_ relaxation rate measurements of 150 µM Magnevist in 100 µM heparin solution after the addition of different ZnCl_2_ concentrations between 125 µM (yellow) and 4 mM (dark red). *R*_1_ values increase after the addition of ZnCl_2_ at time point 0 and reach new plateau values that increase with increasing [ZnCl_2_]. The time constant for the observed transchelation process decreases from more than 21 days for [ZnCl_2_] ≤ 0.5 mM to less than 2 h for [ZnCl_2_] = 4 mM (c.f. Table 1). (**B**) Final *R*_1_ values (new chemical equilibria) as a function of [ZnCl_2_] for Magnevist (red triangles) and Dotarem (black circles). In contrast to Magnevist, no changes of *R*_1_ as a function of [ZnCl_2_] were observed for Dotarem. The red solid line represents the fit using a logistic function (fit results are displayed). The physiological range of Zn^2+^ concentrations is labelled in green. (**C**) Bar charts of the individual contributions of water (blue), of the intact GBCA (yellow), of heparin (green), and of formed Gd-heparin complexes (red) to the overall observed relaxation rate of 150 µM Magnevist solution 5 days after the addition of different ZnCl_2_ concentrations. (**D**) Same as (**C**) but for Dotarem instead of Magnevist.

Due to the negligibility of the term ${R}_{1,\mathrm{Gd\, in\, solution}}$ in Eq. (3), the amount of Gd^3+^ ions that transchelate from a GBCA to the sufficiently large pool of alternative chelators after the addition of a specific amount of competing ions can be quantified using Eq. (5). Similar to the transmetallation experiments in water (Fig. 3), we exemplarily measured the relaxation rate of an aqueous solution containing 100 µM heparin and 150 µM Magnevist as a function of time after the addition of six different ZnCl_2_ stimuli between 0.125 and 4 mM (Fig. 5A). The time constants for the transchelation process significantly differ from the time constants for the transmetallation process in nanopure water. While the latter were on the order of 2 min, the time constants for the transchelation are on the order of hours to weeks and tremendously decrease with increasing ZnCl_2_ concentrations. The kinetic stability in the presence of heparin is 284-fold reduced when [Zn^2+^] increases eightfold from 0.5 to 4 mM. Sub-mM Zn^2+^ stimuli are still associated with a high kinetic stability.

However, in all cases, the final equilibrium *R*_1_ values increase with increasing ZnCl_2_ concentrations as shown in Fig. 5B. While no change of *R*_1_ is observed for Dotarem, *R*_1_ values for the Magnevist sample increase (ca. fourfold) with increasing ZnCl_2_ concentrations and reach a plateau for [ZnCl_2_] > 8 mM. Solving Eqs. (5) and (4) for all measured *R*_1_ values, allows determining the individual contributions of water (${R}_{1, \text {H}_{2} \text {O}}$), the added heparin (${R}_{1,\text {heparin}}$), the intact GBCA (${R}_{1,\text {GBCA}}$), and the Gd^3+^ ions that transchelated to heparin (${R}_{1, \text {Gd@heparin} }$) to the total relaxation rate. The corresponding bar charts (Fig. 5C and D) with the disentangled contributions from *r*_1_^I^, *r*_1_^II^, *r*_1_^III^ illustrate even more clearly that a ZnCl_2_ concentration of 4 mM leads to an almost complete transchelation of the Gd^3+^ ions from Magnevist to heparin, while no transchelation is observed for Dotarem.

The data points for Magnevist are fitted again using a logistic function. The determined ZnCl_2_ concentration needed to dissociate 50% of the Magnevist in heparin solution is (1.86 ± 0.12) mM. This corresponds to a 12.4-fold excess of ZnCl_2_ compared to Magnevist and is therefore about 4.35 times smaller compared to the ~ 54-fold excess that was needed in nanopure water. The combined stimulus of Zn^2+^ and GAG clearly reduces the thermodynamic stability of Gd-DTPA compared to Zn^2+^ alone. The kinetics, however, are slow as the time constant for reaching an equilibrium with a 2 mM Zn^2+^ stimulus is ~ 9.8 h compared to 2.3 min in the absence of heparin. For any given Zn^2+^ stimulus, the kinetic stability is much lower without GAG. However, the ratio of the reaction time constants towards equilibrium, ε = τ_w/GAG_/τ_w/oGAG_, decreases dramatically (440-fold) within a relatively small range of 0.5 to 4 mM (supp. Mat. Fig. S2). In general, the findings for the transmetallation, the binding to heparin, and the transchelation process at 37 °C are in qualitative agreement with the observations at 25 °C.

**Figure 6:**
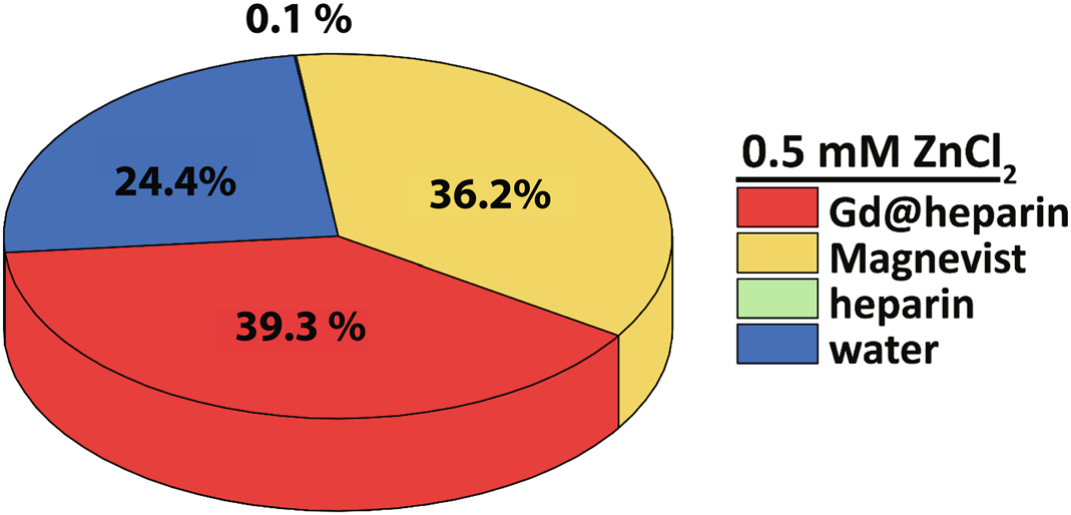
Pie chart of the individual contributions of water (blue; $R_{{1, \text {H}_{2} \text {O}}}$), the intact Magnevist (yellow; $R_{1,\text {GBCA}}$), heparin (green; $R_{1, \text {heparin}}$), and of formed Gd-heparin complexes (red; $R_{1, \text {Gd@heparin}}$) to the overall observed relaxation rate after a stimulus of 0.5 mM ZnCl_2_ at 9.4 T and 25 °C. Although just ~ 15% of the Gd^3+^ ions transchelated to heparin (c.f. Fig. 5B), their contribution (39.3%) already exceeds the contribution (36.2%) of the remaining 85% of intact Magnevist.

Finally, Fig. 6 shows a pie chart of the data for 0.5 mM ZnCl_2_ from Fig. 5C to better visualize the fractional contributions of the individual relaxation rates. At the high physiological concentration of 0.5 mM Zn^2+^, about 15% of the Gd^3+^ ions transchelate to heparin (c.f. Fig. 5B), but the contribution of these few high-relaxivity macromolecular compounds already exceeds the contribution of the remaining 85% of intact GBCA. While it will take several weeks for this reaction to reach chemical equilibrium under the conditions used here, the limiting factor, all things considered, is clearly the pre-occupation of heparin by the Zn^2+^ excess. This becomes evident when a reduced amount of heparin is used and the kinetics speed up significantly for the same Zn stimulus (see supp. Mat. Fig. S3).

## Discussion

In this study, we showed that the quantification of (i) the transmetallation of GBCAs, (ii) the binding of Gd^3+^ ions to an alternative chelator, and (iii) of the combined transchelation process of released Gd^3+^ into competing chelator complexes is possible by means of NMR relaxometry in a time-resolved manner. A key feature is that the different states of Gd, i.e., in the parent GBCA structure, as free ions, and as biointerface-bound ions, can be assessed by rather discrete relaxivities. This yields new insights into both thermodynamic and kinetic stability and allows studying changes in kinetics as well as identifying the certain rate limiting steps. Moreover, the results illustrate an important double role of competing chelators like GAGs: firstly, they reduce the thermodynamic stability of GBCAs in vitro by sequestering Gd^3+^ from the disfavored ZnL + Gd^3+^ intermediate. At the same time, the GAGs interaction with competing ions can suppress the initial attack and increase the kinetic stability significantly.

It should be mentioned that the species with the highest MW is not necessarily the one with the highest relaxivity. Transchelation to macromolecules can also reduce the relaxivity compared to that of free Gd^3+^^40^. However, the prerequisite for the presented approach is just a change in relaxivity, irrespective of enhanced or reduced relaxivity.

Using MRI, i.e., exactly the method that all GBCAs are designed for, as an analytical tool has several advantages over alternative chemical high-precision measurements. MRI-based approaches enable the simultaneous measurement of many samples at exactly the same experimental conditions and minimize systematic errors. However, besides MRI devices, the method can also be implemented at every NMR spectrometer or benchtop NMR relaxometer (albeit with the need to measure each sample individually) and thus ensures a broad applicability at institutions dealing with research on GBCAs. This might prove useful to further link the in vitro findings with preclinical and clinical in vivo observations, which could help to identify the different components that contribute to the observed hyperintensities in various tissues. Due to the dependency of relaxivity values on field strength and temperature^2,38^, the relaxivities of the individual components need to be quantified before the presented approach is applied under different conditions. In general, *R*_2_ relaxometry enables the quantification of transchelation as well. However, since typical *R*_2_ values are about one order of magnitude larger compared to *R*_1_, relative changes are expected to be smaller and thus less suitable.

Well-defined sample solutions enable the discrimination of the different contributions and the equilibrium shifts among the different pools illustrated in Fig. 1b. While the conditions in these simplified sample solutions are obviously less complex than the in vivo situation (e.g., rotational tumbling of newly formed in vivo species is hard to guess), it is the only option to isolate and identify the contributions from different possible molecular components and to eliminate interfering background signals.

The used contrast agents Magnevist (Gd-DTPA) and Dotarem (Gd-DOTA) were chosen as representative examples for a linear and a macrocyclic GBCA, respectively. Gd-DOTA is still clinically used and both complexes play an important role as building blocks for designing novel reporters^41–44^. However, our method can be applied to all available MRI contrast agents (with various paramagnetic ions) and their respective building blocks. The same applies for the utilized heparin, which can be replaced by any other GAG (e.g., chondroitin sulfate) or, more general, by any macromolecular complex that features binding sites for cations. Applying the proposed approach to quantify and compare all clinically approved GBCAs and all possible macromolecular chelator structures is far beyond the scope of this methodical manuscript. However, this should be done in forthcoming studies because the identification of substances to which the released Gd^3+^ ions link in the body and the determination of their NMR properties is still considered one of the most important tasks in this field^45,46^.

Besides GAGs like heparin that we investigated in this study, other substances like transferrin, albumin and citrate are conceivable binding partners that are currently under investigation^47^. However, due to their distribution, their macromolecular size, and their chelating capacity, GAGs are one of the prime candidates for the binding process of Gd^3+^ ions. GAGs are an elementary part of the human glycome with a great structural diversity and a high physiological significance. They appear in the extracellular matrix, on cell surfaces, and in cells with local hotspots throughout the body^48^. Furthermore, due to the participation of GAGs as both pro-inflammatory and anti-inflammatory mediators^49^ and the fact that plasma levels of GAGs are known to be increased in patients with advanced renal insufficiency^50^, GAGs have already been discussed as trigger of inflammatory processes as in nephrogenic systemic fibrosis (NSF), which itself has been linked to the deposition of Gd after the administration of (linear) GBCAs^51–53^. With our measurements, we could confirm the known fact that linear GBCAs are more likely to be transmetallated by zinc than macrocyclic ones^13,31^.

By going beyond the physiological zinc concentration, which reaches up to several hundred micromolar^54–56^, we were able to reach a complete exchange of the paramagnetic central ion for Magnevist and to a significant extend also for Dotarem. Using a large concentration range including (unphysiologically) high concentrations substantially increases the fitting quality and robustness as the system comprises both different chemical equilibria. Theoretically, a robust fit even allows extrapolation and thus the determination of the amount of dissociated gadolinium ions for zinc concentrations, where the transmetallation-induced relaxivity changes are below the limit of detection und would therefore otherwise be elusive for NMR-based investigations. However, such extrapolations should be verified in forthcoming studies using high precision measurements like inductively coupled plasma mass spectrometry^23,28^ or micellar electrokinetic capillary chromatography (MEKC)^57^. Importantly, similar quantifications as for the presented zinc-induced transmetallation can be performed for any competing ions as representatively shown for CaCl_2_ in the supplementary materials. Ions like Fe^2+^ and Cu^2+^ are known to lead to a transmetallation of GBCAs as well^4,13^ and should thus be investigated and quantified in future studies using the proposed approach as well.

The quantitative experimental results regarding the transchelation of Gd ions from GBCAs to macromolecular chelators like GAGs reveal several important new aspects. We could show that a major decomposition of the parent GBCA only occurs upon further impact on the (ZnL + Gd^3+^) intermediate. Importantly, additional Zn^2+^ for completing the transmetallation process and a Gd^3+^ sequester play a synergistic role. GAGs are of twofold interest as their Gd^3+^ binding capacity reduces the thermodynamic stability but comes along with a Zn^2+^ storage capacity that has an opposite effect and leads to an essential increase of the kinetic stability.

For the in vitro experiments, this Zn-heparin interaction has important consequences that help to decipher the complex interplay: The increased kinetic stability in the presence of GAG can have two explanations. Either the initial attack is suppressed by withholding Zn^2+^ or the released Gd^3+^ is very slow to bind into the GAG matrix. The GdCl_3_ + GAG measurements showed an instantaneous equilibrium; thus, the latter explanation would only hold if bound Zn^2+^ blocks the Gd^3+^ to enter the GAG matrix. However, the thermodynamic stability does not support this argument because a 33-fold excess of Zn shifted the point of inflection for Gd binding (Fig. 4) only marginally. This is in agreement with previous studies, which showed that Gd^3+^ outcompetes other endogenous divalent (e.g. Ca^2+^) and monovalent (e.g. K^+^, Na^+^) ions^32^. Moreover, as shown in the supplemental information, faster kinetics are easily regained for reduced heparin concentration. Thus, we conclude that the explanation must be a suppression of the initial attack by withholding Zn^2+^.

It is remarkable that the affinity of Gd^3+^ for heparin is barely affected by large amounts of Zn^2+^, while a relatively small heparin pool is highly efficient in obstructing the attack by a large Zn^2+^ pool. Figure 4 shows that at a Gd^3+^/GAG ratio of 1 / 0.3 practically all Gd^3+^ is bound to the matrix. However, 100 µM heparin in Fig. 5 (which shall bind up to 300 µM Gd^3+^) is very efficient in disturbing the attack by 4 mM of Zn^2+^ or even more. Either heparin binds a huge amount of Zn^2+^ (i.e., territorially binding as a long-range electrostatic interaction with full hydration of Gd^3+^ unrestricted mobility *vs*. a site-specific chelation including a fixed number of coordinating groups resulting in lower hydration numbers) or the entire Zn pool is in frequent contact with the GAG such that the average contact time to attack Gd-DTPA is not sufficient to initiate the transchelation.

We showed that the Zn/GAG ratio is critical to regain faster exchange kinetics. Although more Zn^2+^ ions potentially reduce the availability of binding sites in heparin, we observed that the overall transchelation process is significantly more efficient for a high compared to a low Zn/GAG ratio. Our explanation is that the larger ZnCl_2_ concentration causes on average more Zn^2+^ to be outside the GAG and thus encounter GBCA complexes with a subsequent quick transmetallation step. Due to the above-mentioned fact that Gd^3+^ outperforms Zn^2+^ in terms of binding to the GAG, the released Gd^3+^ ions are apparently not limited in finding a binding site and forming the macromolecular Gd-GAG complexes.

Regarding the probability of a Zn-induced GBCA destabilization in clinical practice, the in vivo conditions are obviously more complex. 75% of serum Zn^2+^ ions are bound to albumin. However, this “inaccessible” pool is physiologically important, and its discharge can be related to problems in homeostasis with noticeable consequences like blood coagulation^36^. Proteins also serve to bind Zn^2+^ in vivo, presumably via glycine and cysteine residues. However, these amino acids have surprisingly little effect on the catalytic function for attacking Gd bound to DTPA^17^ and the “inactivation” effect through such binding should not be overestimated.

Once the Zn-induced destabilization occurs on the GBCA, we could confirm that the subsequent Gd-GAG complexes have an extremely high relaxivity for bulk water protons and we have determined this value with high accuracy. These high relaxivity contributions should be further investigated, because it was conceived that the observed long-term MR signal enhancements in vivo after GBCA administration must result from contributions of intact GBCA complexes, but also from additional soluble gadolinium-containing macromolecular species that are characterized by high relaxivity values^29,30^. Even small concentrations of Gd complexed by GAGs have a huge effect on the observed relaxation rate (c.f. Fig. 6) and could thus readily explain the fact that hyperintensities can be observed in *T*_1_-weighted images in vivo even for trace amounts of Gd long after *i.v.* injection of the GBCA^15,18,20,22–28^.

The high relaxivity can theoretically be caused by multiple contributing factors, as also used in the design of contrast agents that rely on switchable relaxivity^58,59^. The most obvious ones are the reduced intra-molecular mobility^60^ and the reduced overall molecular tumbling of GAG-associated Gd^3+^ ions due to the large molecular weight of the GAG. The tumbling rate is closer to the Larmor frequency at high magnetic field strength and thus lead to more effective relaxation. In addition to the longer rotational correlation time (τ_R_) as a consequence of the reduced rotational tumbling, this could also influence the water residence lifetime at the Gd^3+^ ion, τ_m_, and the water diffusional correlation time, τ_D_. Increased *r*_1_ relaxivities due to changes in these correlation times have been reported for heparin-stabilized iron oxide (Fe_3_O_4_) nanoparticles^61^ and related approaches such as stabilization of Fe_3_O_4_^62^ or cobalt ferrite (CoFe_2_O_4_)^63^ with the polyelectrolyte PSSS (poly(sodium 4-styrenesulfonate)) as well as phospholipid-coated Fe_3_O_4_^64^. Furthermore, the observed relaxivity strongly depends on the number of water molecules coordinated to the Gd^3+^ ions and the presence of second sphere water molecules^65,66^ can influence the relaxivity, as well.

Summarized, we could demonstrate that the proposed dynamic NMR relaxometry-based approach, which can easily be used at every spectrometer and clinical or experimental MRI system, enables the quantification regarding equilibria concentrations and kinetics of (1) the ion-induced transmetallation of GBCAs, (2) the binding of Gd^3+^ ions to macromolecular structures like GAGs, and (3) the combined transchelation process of Gd^3+^ ions from GBCAs to such macromolecules. Thus, the approach can be used to identify the most effective competing ions leading to transmetallation and the most potent Gd-complexing macromolecular species as well as to eliminate less potent candidates and combinations. The presence of competing chelators was shown to have both stabilizing and destabilizing effects on GBCAs, and the overall homeostasis can be a critical tipping point for thermodynamic and kinetic aspects. The macromolecular Gd-GAG complexes with high relaxivity could have a notable influence on observed relaxation times and image contrast even at low Gd^3+^-concentrations. In forthcoming studies, all endogenously occurring competing ions as well as endogenously occurring macromolecular substances that might act as competing chelators should be quantified and compared using the presented NMR-based approach.

## Methods

### Materials

GdCl_3_ (Gadolinium(III)chloride hexahydrate, 99% titration) and ZnCl_2_ (zinc chloride *puriss*.) served as source for free ions (both salts purchased from Sigma‐Aldrich Chemie GmbH, Steinheim, Germany). As example for a human endogenous GAG, a commercially available heparin solution (Heparin‐Natrium‐250,000‐ratiopharm, 250,000 IU/mL, average molecular weight MW = 13 kDa, Ratiopharm GmbH, Ulm, Germany) was used.

Magnevist (Gd-DTPA, Bayer Vital; Leverkusen, Germany) and Dotarem (Gd-DOTA, Guerbet; Sulzbach, Germany) served as representative linear and macrocyclic low-molecular weight GBCA, respectively. Nanopure water (18 MΩ cm) was used for the preparation of model solutions. All employed chemicals were utilized as received without any further purification.

### NMR relaxometry measurements

All experiments were performed on a 9.4 T micro-imaging MR system (Bruker Biospin, Ettlingen, Germany) using a 25 mm double-resonant ^1^H/^129^Xe coil (^129^Xe coil not used). Measurements of the ^1^H *T*_1_ relaxation time were performed using a saturation/dephasing recovery pulse sequence consisting of 50 non-selective π/2 pulses with interleaved gradient spoiling followed by a varying recovery delay (*t*_rec_) and a subsequent gradient echo (GRE)-based, centric-reordered image readout. Image readout parameters were: FoV = 20 × 20 mm^2^, matrix = 128 × 128, slice thickness = 2 mm, *BW* = 50 kHz, *TE* = 2.5 ms, *TR* = 5.7 ms. A variable temperature unit was used to control the sample temperature. If not mentioned otherwise, examinations were performed about 1 h after the sample solutions were put into the magnet to ensure a stable temperature of either 25 °C or 37 °C. All measurements were performed using custom-built tube holders that fit either 7 (*d* = 5 mm) or 16 (*d* = 2.5 mm) NMR tubes to enable the simultaneous measurement of multiple sample solutions under identical experimental conditions. The sample holder was inserted into a 25 mm NMR tube and the void space between the individual sample tubes was filled with Fluorinert for improved susceptibility conditions.

Quantitative *R*_1_ (1/*T*_1_) values were determined by fitting a mono-exponential function to the region of interest (ROI)-averaged data obtained for a set of different *T*_1_-weighted images resulting from varying recovery times between 10 ms and 6 s. The total acquisition time for a *T*_1_ map is given by ${t}_{\mathrm{total}}=\sum_{i}^{N}{t}_{\mathrm{rec},i}+N\cdot ({t}_{\mathrm{sat}}+{t}_{\mathrm{img}})$, where *N* is the number of repetitions, *t*_sat_ is the time needed for the non-selective saturation and *t*_img_ is the acquisition time of a single image (~ 730 ms). All values shown represent the average (± 1 SD) of 10 independently acquired *T*_1_ maps.

### *r*_1_ determinations

The relaxivities (*r*_1_ [s^-1^ mM^-1^]) of both GBCAs, as well as of GdCl_3_, ZnCl_2_, and heparin were determined in nanopure water. Further, the relaxivities of both GBCAs, GdCl_3_, and ZnCl_2_ were determined in 100 µM heparin solution. For obtaining each *r*_1_ value, the relaxation rates (*R*_1_) of six different samples were plotted as a function of the compound concentration and fitted using a linear model. The determined relaxivities and the concentrations used for the individual compounds are listed in supplemental Tables S1 and S2, respectively. All quantifications were done at 25 °C and at 37 °C.

### NMR titration experiments

NMR titration experiments were performed to investigate the binding of Gd^3+^ ions to heparin using two sets of 16 sample solutions, each. The sample solutions consisted of different heparin concentrations between 2.5 nM and 250 µM (based on average MW) mixed with 25 µM GdCl_3_ and, for the second set, additionally with 833 µM of ZnCl_2_. The exact concentrations are listed in supplemental Table S3.

### Transmetallation experiments

To quantify the amount of released Gd^3+^ ions from GBCAs due to the transmetallation with Zn^2+^, 150 µM Magnevist or Dotarem were mixed with 13 different ZnCl_2_ concentrations ranging from 0 to 256 mM (supplemental Table S4). All samples were stored at 25 °C for 4 days to ensure that the transmetallation process was in a steady state. Additional time resolved *T*_1_ measurements were performed for a subset of 7 samples. The amount of Gd^3+^ ions released into water can be quantified by solving a simple linear equation. The observed relaxation rate ($R_{1, \text {obs}}$) after the transmetallation process is given by:

1 $$\begin{aligned} R_{1, \text {obs}} & = R_{{1,\text {H}_{2 \text {O}} }} + R_{1, \text {GBCA}} + R_{{1, \text {GdCl}_{3} }} + R_{{1, \text {ZnCl}_{2} }} \\ & \begin{array}{*{20}c} { = R_{{1, \text {H}_{2} \text O}} + \left( {c_ \text {GBCA} - x} \right) \cdot r_{1,\text {GBCA}} + x \cdot r_{{1, \text {GdCl}_{3} }} + c_{{\text {ZnCl}_{2} }} \cdot r_{{1,\text {ZnCl}_{2} }} } \\ \end{array} \\ \end{aligned}$$

$c_{\text {ZnCl}_{2} }$ is the concentration of ZnCl_2_, $c_\text{GBCA}$ is the starting concentration of the utilized GBCA (here: 150 µM), and $r_{1, \text {GBCA}}$, $r_{{1,\text {GdCl}_{3} }}$, and ${r}_{1,\text {ZnCl}_{\mathrm 2} }$ are the relaxivities of the GBCA, of GdCl_3_, and of ZnCl_2_, respectively. Equation (1) can be solved for *x*, i.e. the concentration of released Gd^3+^ ions reads:

2 $$x = \frac{{R_{1,{\text {obs}}} - R_{{1,{\text {H}}_{2} \text {O}}} - c_{\mathrm{GBCA}} \cdot r_{1, {\text {GBCA}}} - c_{{{\text {ZnCl}}_{2} }} \cdot r_{{1, \text {ZnCl}_{2} }} }}{{r_{{1, {\text {GdCl}}_{3} }} - r_{1,\text {GBCA}} }}  $$

### Transchelation experiments

To quantify the increasing amount of Gd^3+^ ions that transchelate from the GBCAs to heparin over time, 150 µM Magnevist or Dotarem were dissolved in a 100 µM heparin solution and mixed with 8 different ZnCl_2_ concentrations ranging from 1/8 mM to 8 mM. To follow the transchelation process dynamically, *T*_1_ measurements were carried out every 5 to 10 min over a total time of 4 days at a stable temperature of 25 °C. For the present experimental conditions, the amount of Gd^3+^ ions that transchelated from the utilized GBCA to heparin can be quantified in a similar way as for the transmetallation. In general, the observed relaxation rate ($R_{1, \text {obs}}$) after the partial transchelation is given by:

3 $$\begin{array}{*{20}c} {R_{1, \text {obs}} = R_{{1,\text {H}_{2 \text {O}} }} + R_{1, \text {heparin}} + R_{1, \text {GBCA}} + R_{1, \text {Gd\,in\, solution}} + R_{{1, \text {ZnCl}_{2} }} + R_{1, \text {Gd@heparin}} } \\ \end{array}$$

Due to the low ZnCl_2_ concentrations in combination with the low relaxivity of ZnCl_2_ (c.f. supplemental Table S1), $R_{{1, \text {ZnCl}_{2} }}$ is below the limit of detection and thus negligible in the transchelation experiments. As shown (c.f. Fig. 4), $R_{1,\text {Gd\, in\, solution}}$ can be neglected as well, because no free Gd^3+^ ions remain in solution under the present conditions. Thus, Eq. (3) simplifies to:

4 $$\begin{array}{*{20}c} {R_{1, \text {obs}} = R_{{1, \text {H}_{2} \text{O}}} + R_{1, \text {heparin}} + R_{1,\text {GBCA}} + R_{1, \text {Gd@heparin}} } \\ \end{array}$$

Substituting the relaxation rates with the products of concentrations and relaxivities in Eq. (4), the amount of transchelated Gd^3+^ ions (*x*) can be calculated as follows:

5 $$ x = \frac{{R_{1, \text {obs}} - R_{{1, \text {H}_{2} \text {O}}} - c_{\mathrm{heparin}} \cdot r_{1, \text {heparin}} - c_{\mathrm{GBCA} }\cdot r_{1, {\text {GBCA}}} }}{{r_{1, {\text {Gd@heparin}}} - r_{1,\text {GBCA}} }}  $$

## Supplementary Information

Supplementary Information.

## Abbreviations

GBCA: Gadolinium-based contrast agent
GAG: Glycosaminoglycan
PoI: Point of inflection
