# Supplementary material for: An NMR relaxometry approach for quantitative investigation of the transchelation of gadolinium ions from GBCAs to a competing macromolecular chelator
Source: Sci Rep. 2021 Nov 5;11:21731. doi: 10.1038/s41598-021-00974-4 (PMC8571392; doi:10.1038/s41598-021-00974-4)
Supplement: Supplementary file 1 — Supplementary Information. [file 41598_2021_974_MOESM1_ESM.pdf]

# Supplementary Material

## An NMR Relaxometry Approach for Quantitative Investigation of the Transchelation of Gadolinium Ions from GBCAs to a Competing Macromolecular Chelator

*Patrick Werner[1,2], Matthias Taupitz[2], Leif Schröder[1,3], and Patrick Schuenke[1,4]*

*1) Leibniz-Forschungsinstitut für Molekulare Pharmakologie (FMP), Molecular Imaging, Berlin, Germany*
*2) Charité – Universitätsmedizin Berlin, corporate member of Freie Universität Berlin, Humboldt-Universität zu Berlin, and Berlin Institute of Health, Department of Radiology, Berlin, Germany*
*3) Deutsches Krebsforschungszentrum (DKFZ), Division of Translational Molecular Imaging*
*4) Physikalisch-Technische Bundesanstalt (PTB), Biomedical Magnetic Resonance, Berlin, Germany*

Figure S1. $CaCl_2$-induced transmetallation of Magnevist.
Figure S2. Deceleration factor as a function of $[Zn^{2+}]$.
Figure S3. Comparison of transchelation kinetics in heparin solution.
Table S1. Relaxivity values of used compounds.
Table S2. Utilized concentrations for relaxivity determinations.
Table S3. Utilized concentrations for titration experiments.
Table S4. Utilized concentrations for transmetallation experiments.

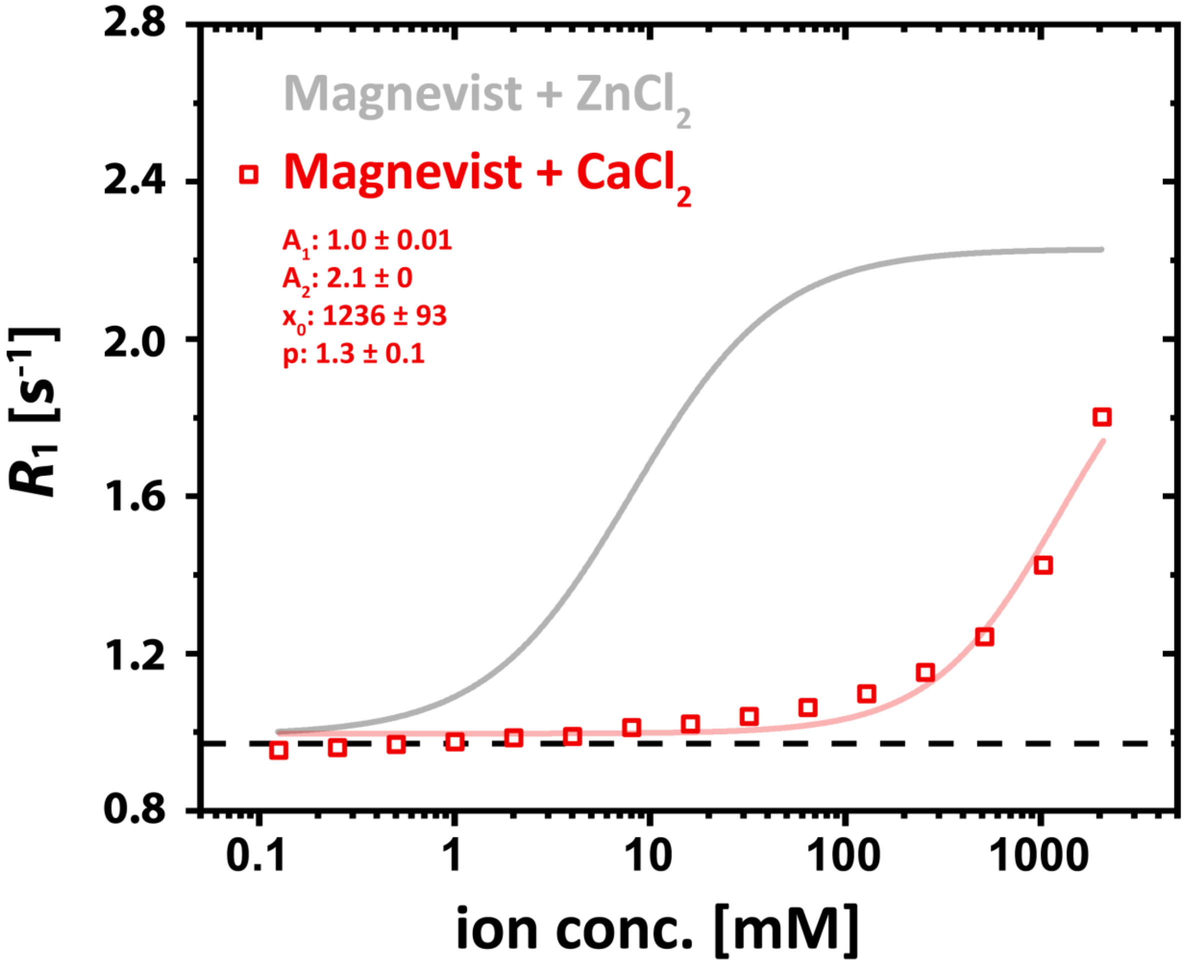


Figure S1: Quantification of the $CaCl_2$-induced transmetallation of 150 µM Magnevist in nanopure water at 9.4 T and 25°C. The red solid line represents the fit using a logistic function (fit parameters are shown; $A_2$ was fixed to the theoretical value of 150 µM $GdCl_3$ in nanopure water). For comparison, the fitted curve of the $ZnCl_2$-induced transmetallation (c.f. Fig. 3B) is shown as gray solid line.

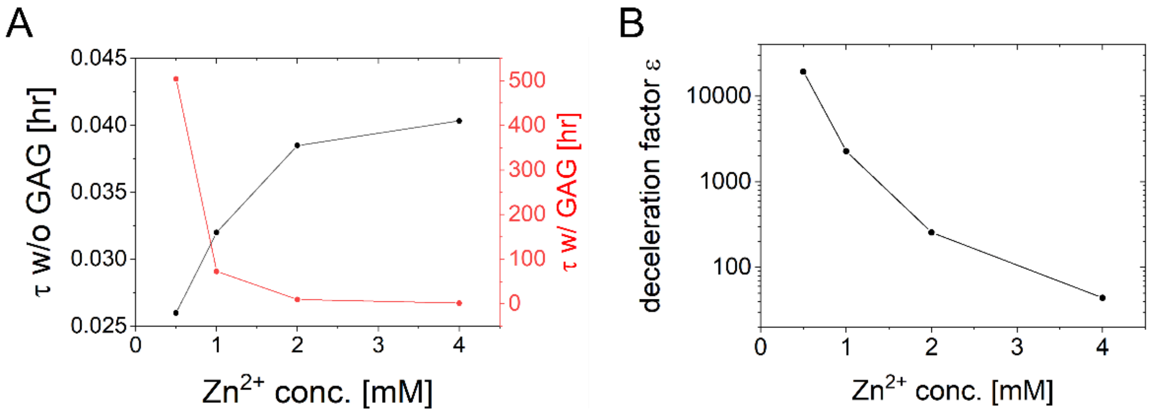


Figure S2: Kinetic stability aspects of transchelation *vs*. transmetallation at 9.4 T and 25°C. **(A)** Comparison of the time constants from Table 1 for the changes in $r_1$ relaxivity representing transmetallation ($ZnCl_2$ + Magnevist; black) or transchelation ($ZnCl_2$ + heparin + Magnevist; red). **(B)** The ratio ε of the time constants with and without heparin from (A) can be interpreted as a deceleration factor that represents the impact of heparin on withholding $Zn^{2+}$ ions from the initial attack.

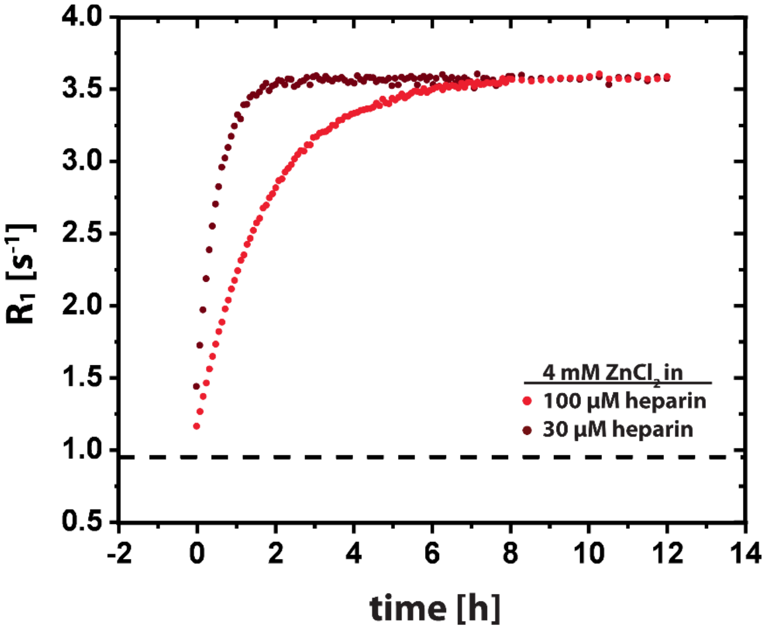


Figure S3: Comparison of transchelation kinetics of 150 µM Magnevist and 4 mM $ZnCl_2$ in 30 µM (dark red) and 100 µM (light red) aqueous heparin solution at 9.4 T and 25°C. The black dashed line represents the theoretical value of 150 µM Magnevist in water. The $R_1$ values of both experiments increase after the addition of $ZnCl_2$ at time point 0 and reach the same new plateau value of $R_1 \approx 3.55\ s^{-1}$ . The time constant for the observed transchelation processes decrease from 1.78 h to less than 35 min when decreasing the heparin concentration from 100 µM to 30 µM.

Table S1: Determined relaxivities (mean ± sd) at 9.4 T of all compounds used in the manuscript. The exact concentrations used for the relaxivity quantifications are listed in supplemental table S2. The relaxivity of $ZnCl_2$ was determined to be smaller than $10^{-4}$ and thus neglected in all calculations.

| | **$r_1$ in $H_20$ [$mM^{-1}$ $s^{-1}$]** | | **$r_1$ in heparin [$mM^{-1}$ $s^{-1}$]** | |
|---|---|---|---|---|
| | 25 °C | 37 °C | 25 °C | 37 °C |
| **Magnevist** | 4.08 ± 0.03 | 3.33 ± 0.03 | 4.04 ± 0.02 | 3.33 ± 0.01 |
| **Dotarem** | 3.72 ± 0.13 | 3.04 ± 0.10 | 3.72 ± 0.01 | 3.06 ± 0.01 |
| **$GdCl_3$** | 11.82 ± 0.13 | 9.16 ± 0.12 | 26.31 ± 0.23 | 25.57 ± 0.27 |
| **$ZnCl_2$** | $< 10^{-4}$ | $< 10^{-4}$ | $< 10^{-4}$ | $< 10^{-4}$ |
| **heparin** | 0.013 ± 0.001 | 0.009 ± 0.001 | - | - |

Table S2: Concentrations used for the determination of the relaxivities ($r_1$) of all compounds used in the manuscript.

| **sample #** | **1** | **2** | **3** | **4** | **5** | **6** |
|---|---|---|---|---|---|---|
| **Magnevist [µM]** | 50 | 100 | 150 | 200 | 300 | 450 |
| **Dotarem [µM]** | 50 | 100 | 150 | 200 | 300 | 450 |
| **$GdCl_3$ [µM]** | 10 | 20 | 40 | 60 | 80 | 100 |
| **$ZnCl_2$ [mM]** | 64 | 128 | 256 | 512 | 1024 | 2048 |
| **heparin [mM]** | 0.5 | 1 | 2 | 3 | 4 | 5 |

Table S3: Concentrations used for the two sets of sample solutions for the titration experiments. Both sets consist of 16 samples, each. One set was prepared with, the other set without the addition of 833 µM $ZnCl_2$.

| **sample #** | **[heparin]/ [$GdCl_3$]** | **$GdCl_3$ [µM]** | **$ZnCl_2$ [µM]** | **heparin [µM]** |
|---|---|---|---|---|
| **1** | 0.0001 | 25 | (833) | 0.00250 |
| **2** | 0.0003 | 25 | (833) | 0.00790 |
| **3** | 0.0010 | 25 | (833) | 0.02500 |
| **4** | 0.0018 | 25 | (833) | 0.04450 |
| **5** | 0.0032 | 25 | (833) | 0.07900 |
| **6** | 0.0056 | 25 | (833) | 0.14050 |
| **7** | 0.0100 | 25 | (833) | 0.25000 |
| **8** | 0.0147 | 25 | (833) | 0.36750 |
| **9** | 0.0215 | 25 | (833) | 0.53750 |
| **10** | 0.0316 | 25 | (833) | 0.79000 |
| **11** | 0.0464 | 25 | (833) | 1.16000 |
| **12** | 0.0681 | 25 | (833) | 1.70250 |
| **13** | 0.1000 | 25 | (833) | 2.50000 |
| **14** | 0.3160 | 25 | (833) | 7.90000 |
| **15** | 1.0000 | 25 | (833) | 25.0000 |
| **16** | 10.000 | 25 | (833) | 250.000 |

Table S4: Concentrations used for the transmetallation experiments.

| sample # | $ZnCl_2$ [µM] | GBCA [µM] |
|---|---|---|
| 1 | 0 | 150 |
| 2 | 0.125 | 150 |
| 3 | 0.25 | 150 |
| 4 | 0.5 | 150 |
| 5 | 1 | 150 |
| 6 | 2 | 150 |
| 7 | 4 | 150 |
| 8 | 8 | 150 |
| 9 | 16 | 150 |
| 10 | 32 | 150 |
| 11 | 64 | 150 |
| 12 | 128 | 150 |
| 13 | 256 | 150 |
